# Low temperature heat capacities and thermodynamic functions described by Debye–Einstein integrals

**DOI:** 10.1007/s00706-017-2117-3

**Published:** 2018-01-25

**Authors:** Ernst Gamsjäger, Manfred Wiessner

**Affiliations:** 1 Institute of Mechanics, Montanuniversität Leoben, Leoben, Austria; 2 Anton Paar GmbH, Graz, Austria

**Keywords:** Calorimetric data, Heat capacity, Debye–Einstein functions, Thermodynamic assessment, Data base

## Abstract

**Abstract:**

Thermodynamic data of various crystalline solids are assessed from low temperature heat capacity measurements, i.e., from almost absolute zero to 300 K by means of semi-empirical models. Previous studies frequently present fit functions with a large amount of coefficients resulting in almost perfect agreement with experimental data. It is, however, pointed out in this work that special care is required to avoid overfitting. Apart from anomalies like phase transformations, it is likely that data from calorimetric measurements can be fitted by a relatively simple Debye–Einstein integral with sufficient precision. Thereby, reliable values for the heat capacities, standard enthalpies, and standard entropies at *T* = 298.15 K are obtained. Standard thermodynamic functions of various compounds strongly differing in the number of atoms in the formula unit can be derived from this fitting procedure and are compared to the results of previous fitting procedures. The residuals are of course larger when the Debye–Einstein integral is applied instead of using a high number of fit coefficients or connected splines, but the semi-empiric fit coefficients keep their meaning with respect to physics. It is suggested to use the Debye–Einstein integral fit as a standard method to describe heat capacities in the range between 0 and 300 K so that the derived thermodynamic functions are obtained on the same theory-related semi-empiric basis. Additional fitting is recommended when a precise description for data at ultra-low temperatures (0–20 K) is requested.

**Graphical abstract:**

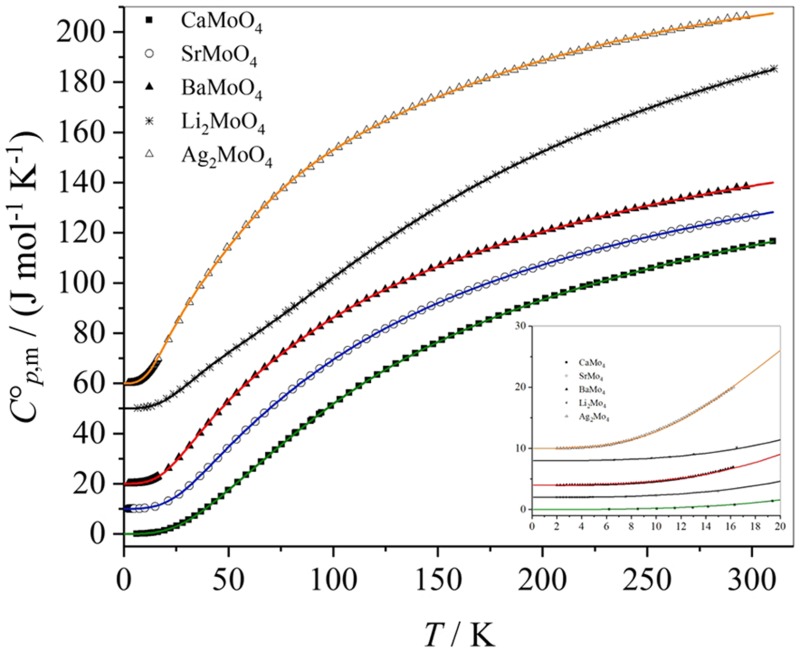

## Introduction

Advanced calorimetric techniques (e.g., by means of a relaxation method instrument [1]) allow for heat capacity measurements at very low temperatures. Recently, heat capacities of various minerals and compounds (e.g., carbonates [2, 3], molybdates [4–8] and Sr-, Rb-, and Cs-substituted barium aluminotitanate hollandites [9]) were determined and the derived thermodynamic functions were reported. Thermodynamic data as provided in Bissengaliyeva et al. [2, 3] are required to describe the formation of lead and zinc carbonates occurring in the oxidation zones of sulfide ore deposits. In addition, theoretical investigations may be assisted by these data when Pb^2+^ ions have to be removed from aqueous solution [10, 11]. Phase stability of molybdates is a key issue when molybdenum containing nuclear waste is immobilized in borosilicate glasses [4–8]. Alternatively, minerals, e.g., barium hollandites [9] that have a robust thermal stability and aqueous durability compared to glass waste forms, are investigated for their potential in hosting radionuclides. Heat capacity measurements and the derived thermodynamic functions are essential for further thermochemical calculations. Currently, heat capacities at temperatures in the range of almost absolute zero to 300 K are fitted by several cubic splines [2, 3] or by more theory-based fitting approaches discriminating low-, mid-, and high-temperature ranges [6, 9]. A combination of Debye–Einstein and Schottky functions are used to fit the temperature-dependent heat capacity data [4, 12] with individual coefficients depending on the temperature region. A general problem common to these fitting approaches is the amount of fit coefficients so that the uncertainty of some fit parameters lies almost in the range of the values of these fit parameters. In addition, the values of these fit coefficients might be strongly correlated. Extending the approach outlined in a previous paper by Gamsjäger et al. [13], it is suggested to use a Debye–Einstein integral fit for the whole temperature range from almost absolute zero to 300 K. It is expected that a unified fitting approach of the heat capacities facilitates the assessment of the thermodynamic data bases.

## Results and discussion

Following Wu et al. [9], the Debye–Einstein integral fit suggested for describing the heat capacities of minerals in the temperature range between 2 and 300 K is defined as:

1 $$C_{{p , {\text{ m}}}}^{\text{o}} = mD(\theta_{\text{D}} /T) + n_{ 1} E_{ 1} (\theta_{\text{E1}} /T) + n_{ 2} E_{ 2} (\theta_{{{\text{E}}2}} /T),$$

with the Debye-Integral $D(\theta_{\text{D}} /T)$:

$$D(\theta_{\text{D}} /T) = 9R(T/\theta_{\text{D}} )^{3} \int\limits_{0}^{{\theta_{\text{D}} /T}} {y^{4} \exp (y)[\exp (y) - 1]^{ - 2} {\text{d}}y,}$$

and the two Einstein terms $E_{1} = E_{1} (\theta_{{{\text{E}}1}} /T)$ and $E_{2} = E_{2} (\theta_{{{\text{E}}2}} /T)$:

$$E_{i} (\theta_{{{\text{E}}i}} /T) = 3R(\theta_{{{\text{E}}i}} /T)^{2} \cdot \exp (\theta_{{{\text{E}}i}} /T) \cdot [\exp (\theta_{{{\text{E}}i}} /T) - 1]^{ - 2} ,$$

where $\theta_{\text{D}}$ is the Debye temperature, $\theta_{{{\text{E}}i}}$ is the Einstein temperature with *i* = 1 or 2 and *m*, *n*_1_, and *n*_2_ are fit parameters. The sum ($m + n_{ 1} + n_{ 2}$) should approximate the number of atoms in the formula unit, see e.g., Wu et al. [9], and Woodfield et al. [14].

The temperature-dependent thermodynamic functions, entropy $S_{\text{m}}^{\text{o}} (T)$ and enthalpy $H_{\text{m}}^{\text{o}} (T)$:

2 $$S_{\text{m}}^{\text{o}} (T) = \int\limits_{0}^{T} {\frac{{C_{{p , {\text{ m}}}}^{\text{o}} (\bar{T})}}{{\bar{T}}}{\text{d}}\bar{T}} ,$$

3 $$H_{\text{m}}^{\text{o}} (T) = \int\limits_{0}^{T} {C_{{p , {\text{ m}}}}^{\text{o}} (\bar{T}){\text{d}}\bar{T}} ,$$

and the derived quantity $S_{\text{m}}^{\text{o}} - H_{\text{m}}^{\text{o}} /T$ are calculated from the $C_{{p , {\text{ m}}}}^{\text{o}}$ (*T*) function by means of Maple 8 [15].

It is worth mentioning that Wu et al. [9] used Eq. (1) for “high T fits” with temperatures higher than 37 K, but not to approximate heat capacity values over the whole temperature range. In addition to “high T fits”, Wu et al. [9] propose a five parameter fit as “low T fit” in the temperature range between 1.9 and 7.5 K and a seven parameter “mid-T fit” between 7.5 and 37 K. The Debye–Einstein integral from Wu et al. [9] is a modification of the semi-empirical approach proposed by Kelley and King [16].

As examples experimental data of heat capacities of carbonates [2, 3], molybdates [4–8], hollandites [9], molybdenum trioxide [17], and a zeolitic polymorph of SiO_2_, faujasite [12], are analyzed by the Debye–Einstein fit approach according to Eq. (1).

### Carbonates

The experimental values of the heat capacities of cerussite (PbCO_3_) provided by Bissengaliyeva et al. [2] exhibit an anomaly at 273 K which is described to be due to water absorbed in the bulk material. The data are fitted according to Eq. (1), where data that deviate due to this anomaly are excluded as their influence on the heat capacity values is marginal. The fit coefficients are calculated by means of the Levenberg–Marquardt algorithm using Origin Pro 2017 [18] and are summarized in Table 1.

**Table 1:** Characteristic temperatures *θ*_D_, *θ*_E1_, *θ*_E2_ and adjusted parameters of the heat capacity of PbCO_3_

Characteristic temperatures	Value	Adjusted pre-factors	Value
*θ*_D_/K	136.2 ± 1.2	*m*	1.446 ± 0.021
*θ*_E1_/K	279.2 ± 2.7	*n*_1_	1.647 ± 0.016
*θ*_E2_/K	1270 ± 16	*n*_2_	1.963 ± 0.042
Number of atoms in formula unit 5		*m* + *n*_1_ + *n*_2_ 5.056	

The temperature-dependent values of the thermodynamic functions provided in [2] are based on cubic polynomials with 36 fit coefficients describing the whole temperature range. It is evident that the uncertainties in the thermodynamic functions are approximately one order of magnitude smaller in case that the $C_{{p , {\text{ m}}}}^{\text{o}}$ (*T*) function is replicated by such a large amount of fit coefficients. However, the semi-empirical Debye–Einstein approach (Eq. (1)) with only six fit coefficients results in very similar temperature-dependent thermodynamic functions.

The uncertainties $\delta C_{{p , {\text{ m}}}}^{\text{o}}$ in the heat capacities $C_{{p , {\text{ m}}}}^{\text{o}}$ are calculated from the standard errors of the fit parameters. The lowest possible value for $C_{{p , {\text{ m}}}}^{\text{o}}$ is subtracted from the highest possible value within these error boundaries and divided by 2 following Eq. (2):

4 $$\delta C_{{p,{\text{ m}}}}^{\text{o}} = \frac{{\left\{ {\begin{array}{*{20}c} {C_{{p,{\text{ p}}}}^{\text{o}} \left[ {m_{\text{p}} ,n_{{1,{\text{p}}}} ,n_{{2,{\text{p}}}} ,\theta_{{{\text{D}},{\text{m}}}} ,\theta_{{{\text{E}}1,{\text{m}}}} ,\theta_{{{\text{E}}2,{\text{m}}}} } \right]} \\ {-C_{{p,{\text{ m}}}}^{\text{o}} \left[ {m_{\text{m}} ,n_{{1,{\text{m}}}} ,n_{{2,{\text{m}}}} ,\theta_{{{\text{D}},{\text{p}}}} ,\theta_{{{\text{E}}1,{\text{p}}}} ,\theta_{{{\text{E}}2,{\text{p}}}} } \right]} \\ \end{array} } \right\}}}{2},$$

with $$m_{\text{p}} = m + \delta m$$, $$n_{{1,{\text{p}}}} = n_{1} + \delta n_{1}$$, $$n_{{2,{\text{p}}}} = n_{2} + \delta n_{2}$$, $$\theta_{\text{D,p}} = \theta_{\text{D}} + \delta \theta_{\text{D}}$$ $$\theta_{\text{E1,p}} = \theta_{\text{E1}} + \delta \theta_{\text{E1}}$$, $$\theta_{\text{E2,p}} = \theta_{\text{E2}} + \delta \theta_{\text{E2}}$$ and $$m_{\text{n}} = m - \delta m$$, $$n_{{1,{\text{n}}}} = n_{1} - \delta n_{1}$$,$$n_{{2,{\text{n}}}} = n_{2} - \delta n_{2}$$, $$\theta_{\text{D,n}} = \theta_{\text{D}} - \delta \theta_{\text{D}}$$, $$\theta_{\text{E1,n}} = \theta_{\text{E1}} - \delta \theta_{\text{E1}}$$, $$\theta_{\text{E2,n}} = \theta_{\text{E2}} - \delta \theta_{\text{E2}} .$$

This approach seems to be safe, results, however, in large uncertainties compared to the uncertainties provided in the literature. The thermodynamic functions of cerussite (PbCO_3_) are calculated and the results are presented in Table 2. The values of the thermodynamic functions at certain temperatures are found to be reasonably close to the values published in Bissengaliyeva et al. [2].

**Table 2:** Thermodynamic functions of PbCO_3_. For each temperature, the thermodynamic data calculated within this study are presented in the first line, and the second line contains data published in Bissengaliyeva et al. [2]

*T*/K	$C_{{p , {\text{ m}}}}^{\text{o}}$ (*T*)/(J mol^−1^ K^−1^)	$S_{\text{m}}^{\text{o}}$ (*T*)/(J mol^−1^ K^−1^)	$H_{\text{m}}^{\text{o}}$ (*T*)/(kJ mol^−1^)	$(S_{\text{m}}^{\text{o}} - H_{\text{m}}^{\text{o}}/T)$/(J mol^−1^ K^−1^)
0	0	0	0	0
50	30.42	18.89	0.6216	6.459
50	30.59 [2]	18.76 [2]	0.6187 [2]	6.383 [2]
100	55.24	48.66	2.8415	20.25
100	55.00 [2]	48.59 [2]	2.8412 [2]	20.18 [2]
150	66.39	73.43	5.914	34.00
150	66.42 [2]	73.26 [2]	5.902 [2]	33.91 [2]
200	73.73	93.56	9.4218	46.45
200	74.07 [2]	93.47 [2]	9.4239 [2]	46.35 [2]
250	80.57	110.75	13.280	57.63
250	80.62 [2]	110.7 [2]	13.288 [2]	57.53 [2]
300	87.05	126.02	17.473	67.78
300	87.30 [2]	126.0 [2]	17.490 [2]	67.69 [2]

The following characteristic temperatures and pre-factors are obtained when applying the Debye–Einstein integral (Eq. (1)) to the heat capacity data of smithsonite [3], see Table 3.

**Table 3:** Characteristic temperatures *θ*_D_, *θ*_E1_, *θ*_E2_ and adjusted parameters of the heat capacity of smithsonite (ZnCO_3_)

Characteristic temperatures	Value	Adjusted pre-factors	Value
*θ*_D_/K	340.1 ± 3.4	*m*	2.018 ± 0.049
*θ*_E1_/K	521 ± 16	*n*_1_	1.134 ± 0.025
*θ*_E2_/K	1174 ± 28	*n*_2_	1.417 ± 0.023
Number of atoms in formula unit 5		*m* + *n*_1_ + *n*_2_ 4.569	

The sum of the pre-factors (*m* + *n*_1_ + *n*_2_) deviates by more than 5% from the number of atoms in the formula unit, but the errors of both the characteristic temperatures and pre-factors are reasonably small again. The thermodynamic data calculated in steps of 50 K are provided in Table 4 and where found to be very similar to those obtained in Bissengaliyeva et al. [3] based on 48 fit coefficients. The temperature-dependent thermodynamic functions are presented in Table 4.

**Table 4:** Thermodynamic functions of ZnCO_3_. For each temperature, the thermodynamic data calculated within this study are presented in the first line; the second line contains data published in Bissengaliyeva et al. [3]

*T*/K	$C_{{p , {\text{ m}}}}^{\text{o}}$ (*T*)/(J mol^−1^ K^−1^)	$S_{\text{m}}^{\text{o}}$ (*T*)/(J mol^−1^ K^−1^)	$H_{\text{m}}^{\text{o}}$ (*T*)/(kJ mol^−1^)	$(S_{\text{m}}^{\text{o}} - H_{\text{m}}^{\text{o}}/T)$/(J mol^−1^ K^−1^)
0	0	0	0	0
50	10.34	3.864	0.143	1.006
50	10.39 [3]	3.759 [3]	0.1415 [3]	0.9285 [3]
100	34.31	18.59	1.275	5.84
100	34.37 [3]	18.49 [3]	1.2736 [3]	5.750 [3]
150	51.58	36.00	3.451	12.99
150	51.60 [3]	35.90 [3]	3.4501 [3]	12.90 [3]
200	63.74	52.59	6.349	20.85
200	63.68 [3]	52.50 [3]	6.3492 [3]	20.75 [3]
250	73.16	67.87	9.781	28.74
250	73.20 [3]	67.73 [3]	9.7794 [3]	28.61 [3]
300	80.63	81.89	13.633	36.45
300	80.60 [3]	81.78 [3]	13.632 [3]	36.34 [3]

**Fig. 1:**
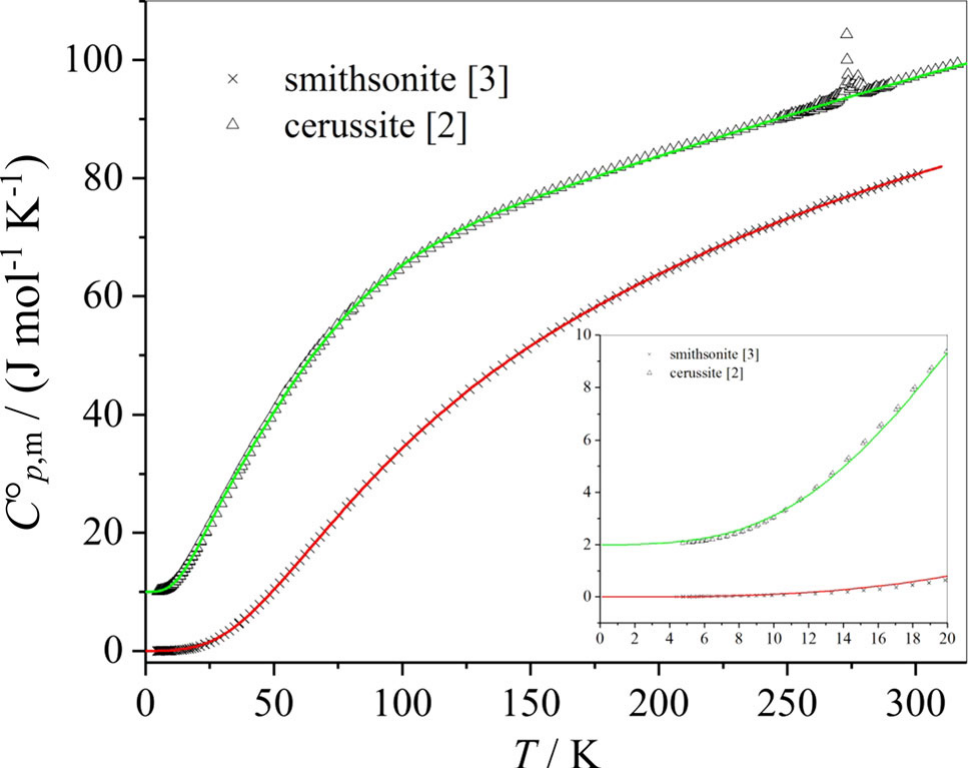
Heat capacities $C_{{p , {\text{ m}}}}^{\text{o}}$ of cerussite and smithsonite and Debye–Einstein fit curves, respectively

Experimental data of the heat capacities of cerussite and smithsonite and Debye–Einstein integral fits according to Eq. (1) are plotted versus temperature in Fig. 1. The heat capacity values of cerussite are shifted upwards by 10 J mol^−1^ K^−1^ (2 J mol^−1^ K^−1^ in the insert) for the sake of a clear arrangement of the curves.

Motivated by the precise thermodynamic data for cerussite and smithsonite based on the simple Debye–Einstein integral fit, the same procedure has been applied to the heat capacities of the molybdates SrMoO_4_, BaMoO_4_, CaMoO_4_, Li_2_MoO_4_, and Ag_2_MoO_4_.

### Molybdates

The characteristic temperatures *θ*_D_, *θ*_E1_, *θ*_E2_ and adjusted parameters to the experimental data of the heat capacity of SrMoO_4_ [4] are presented in Table 5. Thermodynamic functions are provided at several temperatures from 0 to 300 K in Table 6. The fit parameters for the heat capacities of the remaining molybdates that have been analyzed are provided in Table 7 (BaMoO_4_), Table 8 (Ag_2_MoO_4_), Table 9 (Li_2_MoO_4_), Table 10 (CaMoO_4_ [8]), and Table 11 (CaMoO_4_ [17]). The temperature-dependent values of the heat capacities $C_{{p,{\text{ m}}}}^{0}$, entropies $S_{\text{m}}^{\text{o}}$, enthalpies $H_{\text{m}}^{\text{o}}$, and of the derived function $S_{\text{m}}^{\text{o}} - H_{\text{m}}^{\text{o}} /T$ are not provided for the latter molybdates. However, these values can be easily calculated using Eqs. (1)–(3) with the fit coefficients provided in Tables 7, 8, 9, 10 and 11. The absolute values that result from this calculation will again be very close to the values provided in the literature. However, the values at 298.15 K are compared to the literature and presented at the end of this paragraph.

**Table 5:** Characteristic temperatures *θ*_D_, *θ*_E1_, *θ*_E2_ and adjusted parameters *m*, *n*_1_, and *n*_2_ of the heat capacity of SrMoO_4_ [4]

Characteristic temperatures	Value	Adjusted pre-factors	Value
*θ*_D_/K	250.4 ± 1.6	*m*	2.645 ± 0.032
*θ*_E1_/K	470 ± 8	*n*_1_	1.996 ± 0.025
*θ*_E2_/K	1129 ± 29	*n*_2_	1.428 ± 0.023
Number of atoms in formula unit 6		*m* + *n*_1_ + *n*_2_ 6.069

**Table 6:** Thermodynamic functions of SrMoO_4_. For each temperature, the thermodynamic data calculated in this study are presented in the first line; the second line contains data published in Morishita and Houshiyama [4]

*T*/K	$C_{{p , {\text{ m}}}}^{\text{o}}$ (*T*)/(J mol^−1^ K^−1^)	$S_{\text{m}}^{\text{o}}$ (*T*)/(J mol^−1^ K^−1^)	$H_{\text{m}}^{\text{o}}$ (*T*)/(kJ mol^−1^)	$(S_{\text{m}}^{\text{o}} - H_{\text{m}}^{\text{o}}/T)$/(J mol^−1^ K^−1^)
0	0	0	0	0
50	24.61	10.79	0.39	3.02
50	24.27 [4]	10.88 [4]	0.39 [4]	3.08 [4]
100	59.38	39.32	2.55	13.85
100	59.11 [4]	39.18 [4]	2.53 [4]	13.88 [4]
150	81.96	68.00	6.12	27.2
150	81.68 [4]	67.70 [4]	6.09 [4]	27.1 [4]
200	97.14	93.78	10.62	40.7
200	97.71 [4]	93.52 [4]	10.60 [4]	40.52 [4]
250	108.28	116.71	15.77	53.64
250	109.25 [4]	116.65 [4]	15.79 [4]	53.49 [4]
300	116.74	137.24	21.40	65.89
300	116.82 [4]	137.29 [4]	21.46 [4]	65.76 [4]

**Table 7:** Characteristic temperatures *θ*_D_, *θ*_E1_, *θ*_E2_ and adjusted parameters of the heat capacity of BaMoO_4_

Characteristic temperatures	Value	Adjusted pre-factors	Value
*θ*_D_/K	195.5 ± 0.9	*m*	2.488 ± 0.018
*θ*_E1_/K	410.1 ± 4.6	*n*_1_	1.999 ± 0.018
*θ*_E2_/K	1050 ± 18	*n*_2_	1.543 ± 0.017
Number of atoms in formula unit 6		*m* + *n*_1_ + *n*_2_ 6.030

**Table 8:** Characteristic temperatures *θ*_D_, *θ*_E1_, *θ*_E2_ and adjusted parameters of the heat capacity of Ag_2_MoO_4_

Characteristic temperatures	Value	Adjusted pre-factors	Value
*θ*_D_/K	119.0 ± 0.6	*m*	2.379 ± 0.025
*θ*_E1_/K	266.5 ± 3.3	*n*_1_	2.548 ± 0.022
*θ*_E2_/K	769 ± 11	*n*_2_	1.873 ± 0.021
Number of atoms in formula unit 7		*m* + *n*_1_ + *n*_2_ 6.800

**Table 9:** Characteristic temperatures *θ*_D_, *θ*_E1_, *θ*_E2_ and adjusted parameters of the heat capacity of Li_2_MoO_4_

Characteristic temperatures	Value	Adjusted pre-factors	Value
*θ*_D_/K	195.4 ± 1.4	*m*	1.679 ± 0.018
*θ*_E1_/K	471.5 ± 4.4	*n*_1_	3.389 ± 0.040
*θ*_E2_/K	1079 ± 19	*n*_2_	2.419 ± 0.030
Number of atoms in formula unit 7		*m* + *n*_1_ + *n*_2_ 7.487

**Table 10:** Characteristic temperatures *θ*_D_, *θ*_E1_, *θ*_E2_ and adjusted parameters of the heat capacity of CaMoO_4_ (experimental data from [8])

Characteristic temperatures	Value	Adjusted pre-factors	Value
*θ*_D_/K	284.4 ± 1.8	*m*	2.325 ± 0.033
*θ*_E1_/K	465.7 ± 5.1	*n*_1_	2.248 ± 0.017
*θ*_E2_/K	1133 ± 16	*n*_2_	1.565 ± 0.015
Number of atoms in formula unit 6		*m* + *n*_1_ + *n*_2_ 6.138

**Table 11:** Characteristic temperatures *θ*_D_, *θ*_E1_, *θ*_E2_ and adjusted parameters of the heat capacity of CaMoO_4_ (experimental data from [17])

Characteristic temperatures	Value	Adjusted pre-factors	Value
*θ*_D_/K	291.6 ± 2.2	*m*	2.488 ± 0.040
*θ*_E1_/K	497.7 ± 7.3	*n*_1_	2.261 ± 0.022
*θ*_E2_/K	1261 ± 35	*n*_2_	1.562 ± 0.037
Number of atoms in formula unit 6		*m* + *n*_1_ + *n*_2_ 6.311	

**Fig. 2:**
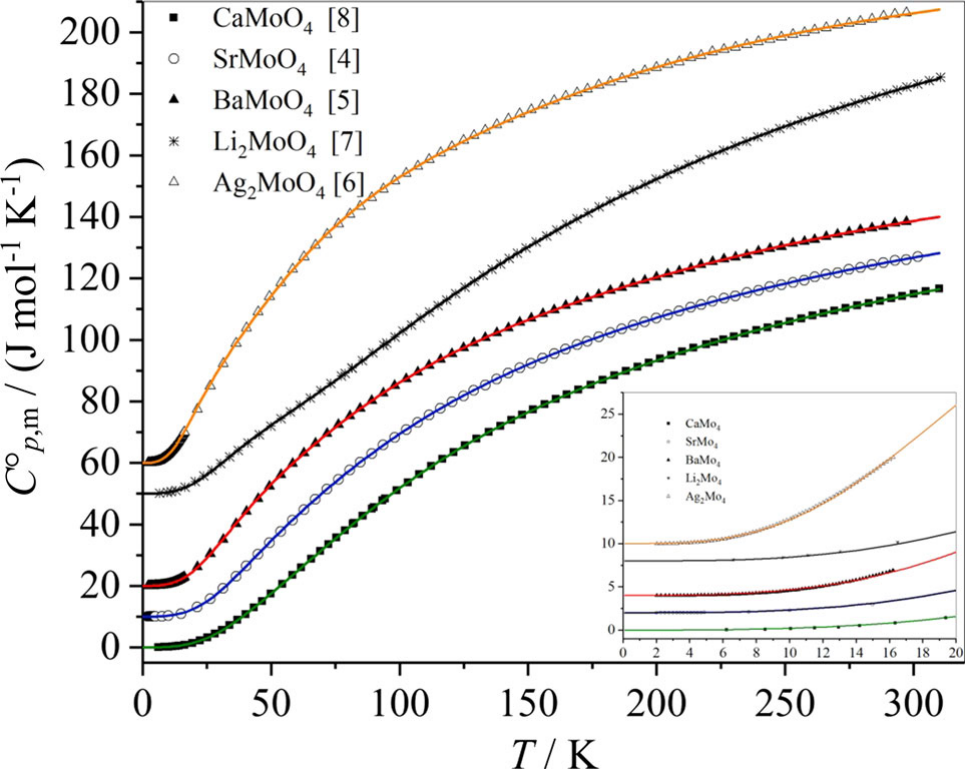
Experimental data of the molar heat capacities of CaMoO_4_, SrMoO_4_, BaMoO_4_, Li_2_MoO_4_, and Ag_2_MoO_4_ and the Debye–Einstein fit curves (data and fit curves below 20 K are shown in the insert)

The experimental data of the temperature-dependent molar heat capacities of the molybdates CaMoO_4_, SrMoO_4_, BaMoO_4_, Li_2_MoO_4_, and Ag_2_MoO_4_ and the Debye–Einstein approximations are presented in Fig. 2. The heat capacities of SrMoO_4_ are shifted upwards by 10 J mol^−1^ K^−1^ (2 J mol^−1^ K^−1^ in the insert), the heat capacities of BaMoO_4_ are shifted upwards by 20 J mol^−1^ K^−1^ (4 J mol^−1^ K^−1^ in the insert), those of Li_2_MoO_4_ by 50 J mol ^−1^ K^−1^ (8 J mol^−1^ K^−1^ in the insert), and those of Ag_2_MoO_4_ by 60 J mol^−1^ K^−1^ (10 J mol^−1^ K^−1^ in the insert) for sake of a clear arrangement in Fig. 2. The comparably small slope of the heat capacities of Li_2_MoO_4_ with temperature at values below 100 K can be explained by the small molar mass of Li_2_MoO_4_ compared to the other molybdates investigated.

### Hollandites

The number of atoms in the unit cell is much higher for Sr-, Rb-, and Cs-substituted barium aluminotitanate hollandites compared to the previously discussed carbonates and molybdates. The characteristic temperatures and the pre-factors of Sr-, Rb-, and Cs-substituted barium aluminotitanate hollandites and their errors are presented in Tables 12, 13, and 14, respectively.

**Table 12:** Characteristic temperatures *θ*_D_, *θ*_E1_, *θ*_E2_ and adjusted parameters of the heat capacity of Sr-substituted barium aluminotitanate hollandite (Ba_1.14_Sr_0.10_Al_2.38_Ti_5.59_O_16_)

Characteristic temperatures	Value	Adjusted pre-factors	Value
*θ*_D_/K	183 ± 7	*m*	2.97 ± 0.21
*θ*_E1_/K	351 ± 14	*n*_1_	7.17 ± 0.35
*θ*_E2_/K	715 ± 11	*n*_2_	14.23 ± 0.44
Number of atoms in formula unit 25.2		*m* + *n*_1_ + *n*_2_ 24.37

**Table 13:** Characteristic temperatures *θ*_D_, *θ*_E1_, *θ*_E2_ and adjusted parameters of the heat capacity of Rb-substituted barium aluminotitanate hollandite (Ba_1.17_Rb_0.19_Al_2.46_Ti_5.53_O_16_)

Characteristic temperatures	Value	Adjusted pre-factors	Value
*θ*_D_/K	190.2 ± 3.2	*m*	3.29 ± 0.11
*θ*_E1_/K	384.6 ± 7.4	*n*_1_	8.69 ± 0.23
*θ*_E2_/K	779.5 ± 8.5	*n*_2_	13.1 ± 0.3
Number of atoms in formula unit 25.4		*m* + *n*_1_ + *n*_2_ 25.08

**Table 14:** Characteristic temperatures *θ*_D_, *θ*_E1_, *θ*_E2_ and adjusted parameters of the heat capacity of Cs-substituted barium aluminotitanate hollandite (Ba_1.18_Cs_0.21_Al_2.44_Ti_5.53_O_16_)

Characteristic temperatures	Value	Adjusted pre-factors	Value
*θ*_D_/K	188.5 ± 3.7	*m*	3.19 ± 0.12
*θ*_E1_/K	376.2 ± 8.2	*n*_1_	8.37 ± 0.25
*θ*_E2_/K	759.2 ± 8.3	*n*_2_	13.8 ± 0.3
Number of atoms in formula unit 25.4		*m* + *n*_1_ + *n*_2_ 25.36	

**Fig. 3:**
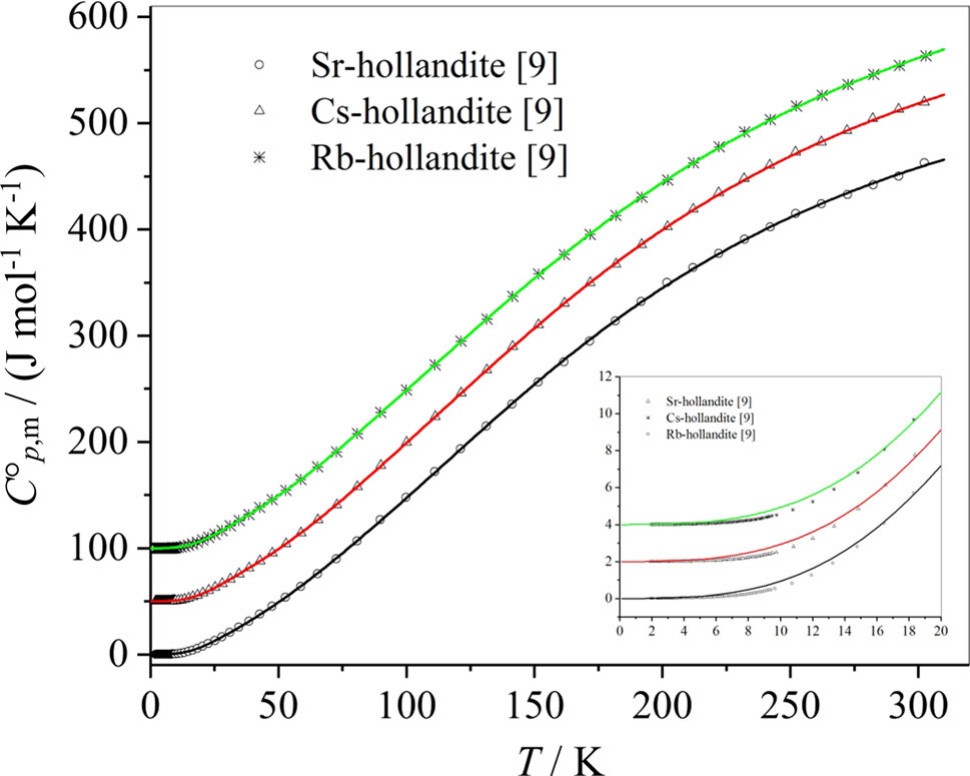
Experimental data of the molar heat capacities of Sr-hollandite, Cs-hollandite, and Rb-hollandite, respectively. The values of the heat capacities of Cs-hollandite are shifted upwards by 50 J mol^−1^ K^−1^, those of Rb-hollandite by 100 J mol^−1^ K^−1^. The experimental data are approximated by Debye–Einstein fit curves according to Eq. (1)

The values for the heat capacities $C_{{p,{\text{ m}}}}^{0}$ fitted by Eq. (1) are again close to the experimental values (Fig. 3), but for the very low heat capacity data a systematic deviation of the fit must be stated.

**Fig. 4:**
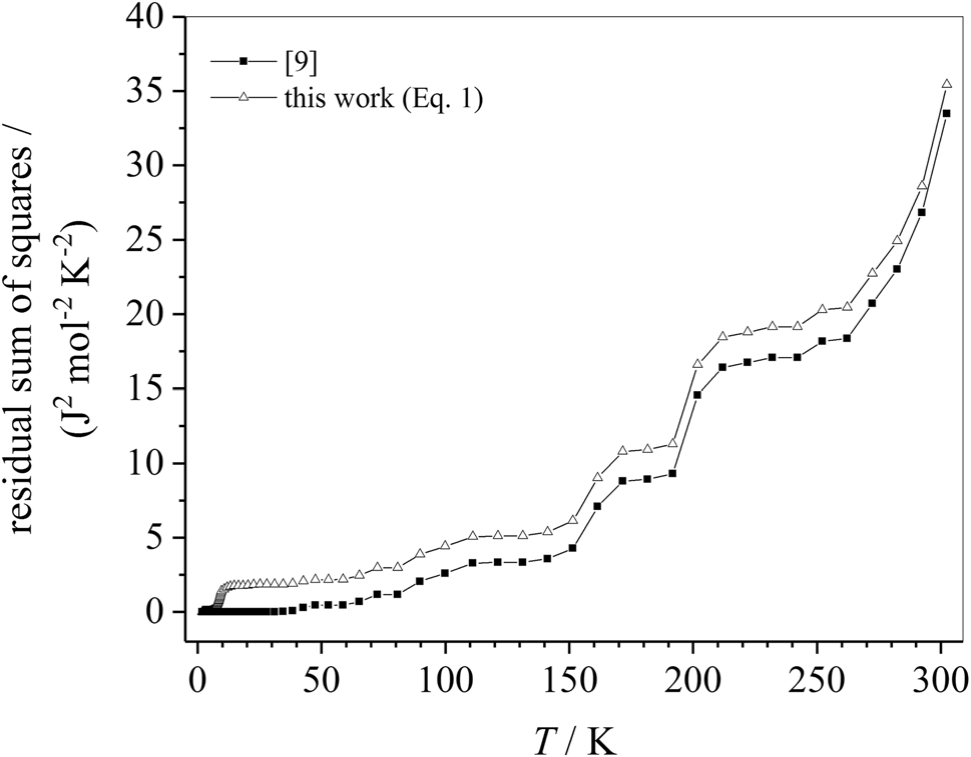
Residual sum of squares from Wu et al. [9] compared to the six parameter Debye–Einstein fit used in this work

However, as both quantities—the heat capacity and the entropy—are close to zero at very low temperatures, the influence of this deviation to the thermodynamic quantities at higher temperatures is in the range of the error of measurement. The quality of this six-parameter Debye–Einstein fit (Eq. (1)) has been compared with the previously published fitting procedure with additional 14-fit parameter in the two temperature ranges also fitted to the data from approx. 2–7.5 K and from 7.5 to 40 K. A quantity relevant for the error of the fit is the residual sum of squares (i.e., sum of the squares of experimental value minus calculated value). It is shown in Fig. 4 that the residual sum of squares is only slightly higher at room temperature for the Debye–Einstein six-parameter fit compared to the fit presented in Wu et al. [9].

The values of the thermodynamic functions of the carbonates, molybdates, and hollandites at 298.15 K are calculated with Eq. (1) and are compared to literature data. These data are compiled in Table 15 for carbonates and molybdates and in Table 16 for hollandites. The first line shows the calculation based on Eq. (1) and the Debye–Einstein fit coefficients, the second line the data from the literature.

**Table 15:** Thermodynamic properties of carbonates (PbCO_3_ and ZnCO_3_), and molybdates (SrMoO_4_, BaMoO_4_, Ag_2_MoO_4_, Li_2_MoO_4_, and CaMoO_4_) at 298.15 K

$C_{{p , {\text{ m}}}}^{\text{o}}$ (*T*)/(J mol^−1^ K^−1^)	$S_{\text{m}}^{\text{o}}$ (*T*)/(J mol^−1^ K^−1^)	$H_{\text{m}}^{\text{o}}$ (*T*)/(kJ mol^−1^)	$(S_{\text{m}}^{\text{o}} - H_{\text{m}}^{\text{o}}/T)$/(J mol^−1^ K^−1^)
Cerussite (PbCO_3_)			
86.4 ± 1.2	125.5 ± 2.4	17.3 ± 0.3	67.4 ± 1.4
87.07 ± 0.09 [2]	125.45 ± 0.20 [2]	17.33 ± 0.02 [2]	67.33 ± 0.18 [2]
Smithsonite (ZnCO_3_)			
80.34 ± 2.3	81.4 ± 3.3	13.5 ± 0.5	36.2 ± 1.6
80.36 ± 0.09 [3]	81.28 ± 0.09 [3]	13.48 ± 0.01 [3]	36.05 ± 0.07 [3]
SrMoO_4_			
116.4 ± 1.9	136.51 ± 3.0	21.2 ± 0.5	65.5 ± 1.5
116.68 [4]	136.56 [4]	21.24 [4]	65.32 [4]
BaMoO_4_			
118.2 ± 1.2	152.6 ± 2.2	22.5 ± 0.3	77.1 ± 1.1
118.47 [5]	152.69 [5]	22.49 [5]	77.26 [5]
Ag_2_MoO_4_			
145.0 ± 1.4	220.2 ± 3.6	29.9 ± 0.5	120.0 ± 2.0
146.20 [6]	219.87 [6]	29.85 [6]	119.8 [6]
Li_2_MoO_4_			
132.14 ± 1.7	135.74 ± 2.9	21.8 ± 0.4	62.6 ± 1.4
132.35 ± 0.21 [7]	135.87 ± 0.21 [7]	21.80 ± 0.04 [7]	62.75 ± 0.15 [7]
CaMoO_4_ (exp. data from [8])			
114.5 ± 1.5	122.1 ± 2.5	19.8 ± 0.4	55.6 ± 1.2
114.61 [8]	122.0 [8]	19.83 [8]	55.54 [8]
CaMoO_4_ (exp. data from [17])			
114.5 ± 2.2	122.2 ± 3.2	19.8 ± 0.5	55.7 ± 1.5
114.31 ± 0.80 [17]	122.23 ± 1.22 [17]	19.83 ± 0.2 [17]	55.71 ± 1.37 [17]

**Table 16:** Thermodynamic properties of Sr-, Rb-, and Cs-substituted barium aluminotitanate hollandites at 298.15 K

$C_{{p , {\text{ m}}}}^{\text{o}}$ (*T*)/(J mol^−1^ K^−1^)	$S_{\text{m}}^{\text{o}}$ (*T*)/(J mol^−1^ K^−1^)	$H_{\text{m}}^{\text{o}}$ (*T*)/(kJ mol^−1^)	$(S_{\text{m}}^{\text{o}} - H_{\text{m}}^{\text{o}}/T)$/(J mol^−1^ K^−1^)
Ba_1.14_Sr_0.10_Al_2.38_Ti_5.59_O_16_			
456.5 ± 9.9	414 ± 20	70.7 ± 3.0	177.0 ± 9.6
456.76 [9]	413.93 ± 8.28 [9]	70.743 ± 0.02 [9]	176.66 ± 0.18 [9]
Ba_1.17_Rb_0.19_Al_2.46_Ti_5.53_O_16_			
459.6 ± 9.5	415 ± 18	70.892 ± 2.8	177.60 ± 8.9
459.96 [9]	415.13 ± 8.30 [9]	70.894 ± [9]	177.35 ± [9]
Ba_1.18_Cs_0.21_Al_2.44_Ti_5.53_O_16_			
467.4 ± 10.5	419.8 ± 20.5	71.88 ± 3.9	178.7 ± 10.0
467.81 [9]	419.59 ± 8.39 [9]	71.888 [9]	178.48 [9]

The values of the thermodynamic quantities for CaMoO_4_ at room temperature due to different low temperature calorimetric measurements ([8] and [17]) are very similar and in both cases the calculated results obtained by the Debye–Einstein fit (Eq. (1)) are very close to the values reported in [8, 17], respectively.

It is worth noting that the thermodynamic quantities for the Sr-, Rb-, and Cs-hollandites at 298.15 K calculated from Eq. (1) and Tables 12, 13 and 14 are very close to the values published in [9], see Table 16. Thus, it is concluded that the error for the heat capacity at ultra-low temperatures (Fig. 3, insert) is small at higher temperatures. The deviations of the thermodynamic values calculated by Eqs. (1)–(3) and the published values are well below the experimental error of about 0.5–0.8% at *T* > 100 K [19].

As stated above, the calculation of the error of the fit by Eq. (4) is rather large (as the complete range of the error of the individual fit coefficients is taken into account) and exceeds the experimental error. However, the absolute values of all thermodynamic data at 298.15 K that have been calculated by means of Eqs. (2) and (3) are sufficiently close at the literature data being within the experimental error.

**Fig. 5:**
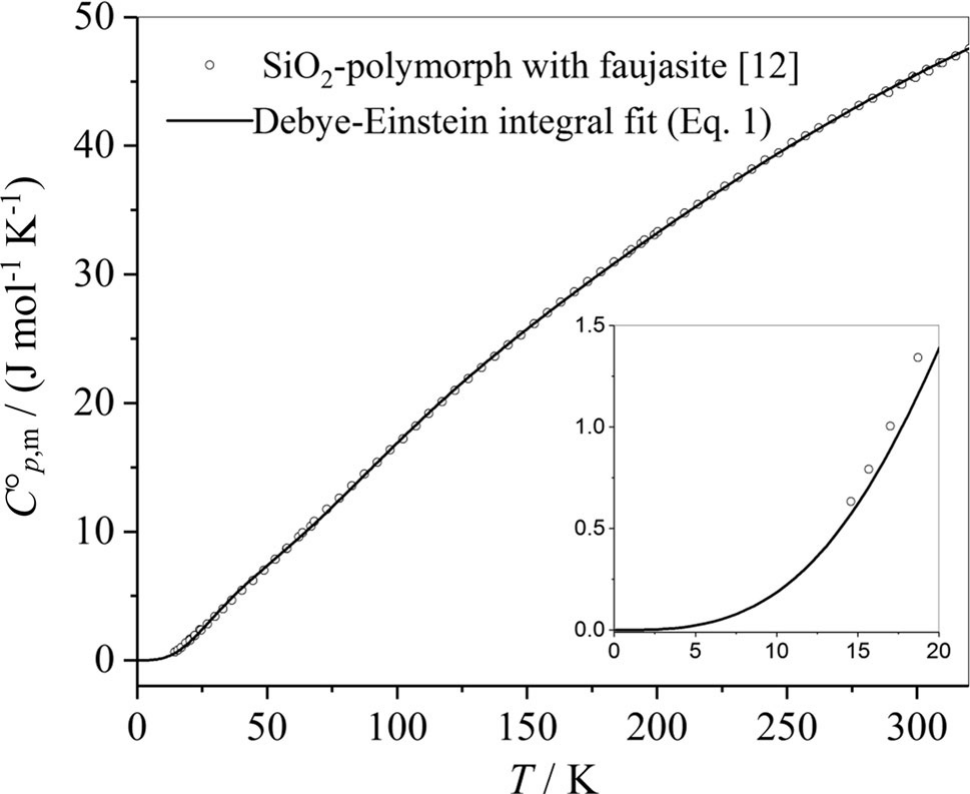
Experimental data of the molar heat capacities of faujasite. The experimental data are approximated by a Debye–Einstein fit curve (the fit slightly deviates from the experimental data at low temperatures)

As a further example for the power of the simple Debye–Einstein approach (Eq. (1)) experimental data of the heat capacities of the SiO_2_ polymorph faujasite [12] are considered. The fit coefficients and the thermodynamic data are presented in Table 17. The heat capacity of the SiO_2_ polymorph faujasite and its Debye–Einstein integral fit, Eq. (1) is plotted versus temperature in Fig. 5.

**Table 17:** Characteristic temperatures *θ*_D_, *θ*_E1_, *θ*_E2_ and adjusted parameters of the heat capacity of the SiO_2_ polymorph with faujasite and thermodynamic properties at 298.15 K

Characteristic temperatures	Value	Adjusted pre-factors	Value
*θ*_D_/K	170.4 ± 2.9	*m*	0.474 ± 0.011
*θ*_E1_/K	439.1 ± 7.2	*n*_1_	1.076 ± 0.016
*θ*_E2_/K	1160.8 ± 15.2	*n*_2_	1.402 ± 0.014
Number of atoms in formula unit 3		*m* + *n*_1_ + *n*_2_ 2.952	

$C_{{p , {\text{ m}}}}^{\text{o}}$ (*T*)/(J mol^−1^ K^−1^)	$S_{\text{m}}^{\text{o}}$ (*T*)/(J mol^−1^ K^−1^)	$H_{\text{m}}^{\text{o}}$ (*T*)/(kJ mol^−1^)	$(S_{\text{m}}^{\text{o}} - H_{\text{m}}^{\text{o}}/T)$/(J mol^−1^ K^−1^)
45.31 ± 0.81	44.79 ± 1.56	7.18 ± 0.2	20.71 ± 0.8
45.34 [12]	44.734 [12]	7.178 [12]	20.658 [12]

The absolute values of the thermodynamic quantities are reasonably close at 298.15 K, when comparing calculation with the very simple Eq. (1) and the values from Boerio-Goates et al. [12]. The six fit coefficients *θ*_D_, *θ*_E1_, *θ*_E2_, *m*, *n*_1_, and *n*_2_ are always valid in the whole temperature range. In the original fit provided in Boerio-Goates et al. [12], a low temperature fit is discriminated from the high temperature fit and an additional Schottky function has been introduced.

**Fig. 6:**
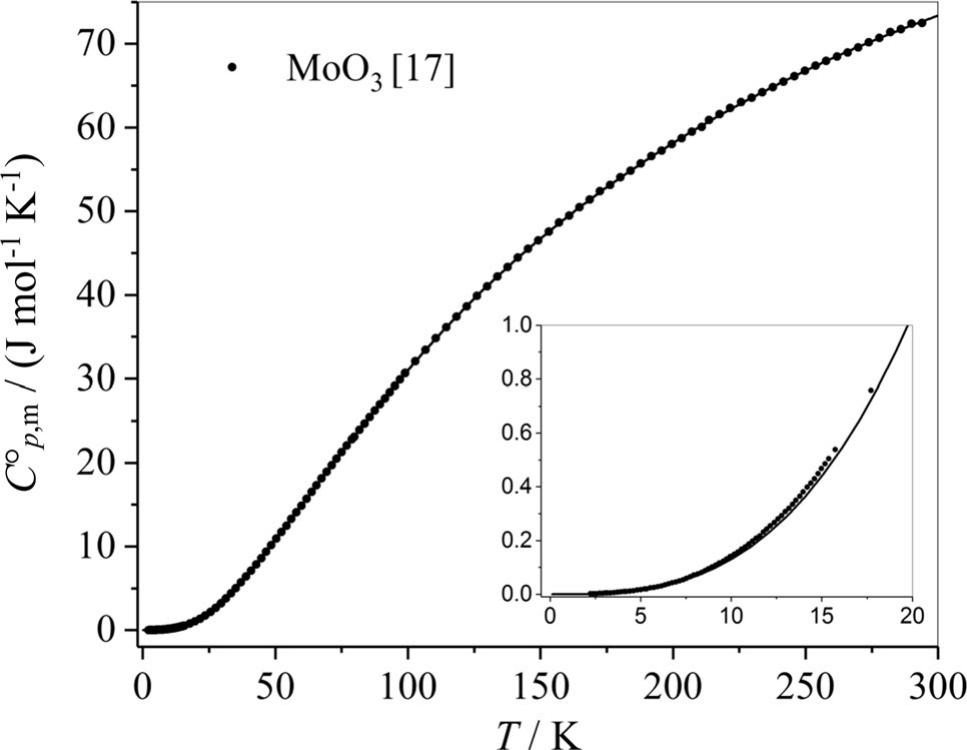
Experimental data of the molar heat capacities of MoO_3_(cr) (data and the fit curve below 20 K are shown in the insert)

Recently, experimental values for the low temperature molar heat capacities of MoO_3_(cr) were published in [17]. Again, the Debye–Einstein approach (Eq. (1)) with only six fit parameters has been used to approximate the molar heat capacities in the temperature range of 2–294 K. The resulting fit coefficients are presented in Table 18 and the comparison between this fit and the experimental data is shown in Fig. 6.

**Table 18:** Characteristic temperatures *θ*_D_, *θ*_E1_, *θ*_E2_ and adjusted parameters of the heat capacity of the MoO_3_(cr) and thermodynamic properties at 298.15 K

Characteristic temperatures	Value	Adjusted pre-factors	Value
*θ*_D_/K	266.7 ± 1.6	*m*	1.271 ± 0.016
*θ*_E1_/K	462.7 ± 4.8	*n*_1_	1.533 ± 0.012
*θ*_E2_/K	1059.8 ± 15.3	*n*_2_	1.184 ± 0.012
Number of atoms in formula unit 4		*m* + *n*_1_ + *n*_2_ 3.988	

$C_{{p , {\text{ m}}}}^{\text{o}}$ (*T*)/(J mol^−1^ K^−1^)	$S_{\text{m}}^{\text{o}}$ (*T*)/(J mol^−1^ K^−1^)	$H_{\text{m}}^{\text{o}}$ (*T*)/(kJ mol^−1^)	$(S_{\text{m}}^{\text{o}} - H_{\text{m}}^{\text{o}}/T)$/(J mol^−1^ K^−1^)
73.1 ± 0.9	75.4 ± 1.5	12.3 ± 0.2	34.05 ± 0.73
73.17 ± 0.71 [17]	75.43 ± 0.75 [17]	12.3 ± 0.1 [17]	34.07 ± 0.86 [17]

It is worth mentioning that the sum of the pre-factors *m*, *n*_1_, and *n*_2_ obeys the Neumann–Kopp rule rather well although not forced by any constraint for all the examples mentioned above.

The problem of overfitting is highlighted by the following example. It has been observed that the mid-range temperature fit for Ba_1.14_Sr_0.10_Al_2.38_Ti_5.59_O_16_ with polynomials containing seven coefficients can be equally well be described by rather different values for the fit coefficients, see Table 19.

**Table 19:** Values of the mid-range temperature fit coefficients for Sr-hollandite according to Wu et al. [9] compared to those obtained in this work

Fit coefficients	Values from Wu et al. [9]	Values and errors, this work
*A*_0_/J mol^−1^ K^−1^	− 1.7075	− 2.76 ± 0.35
*A*_1_/J mol^−1^ K^−2^	0.88203	1.34 ± 0.13
*A*_2_/J mol^−1^ K^−3^	− 0.16900	− 0.248 ± 0.020
*A*_3_/J mol^−1^ K^−4^	0.014863	0.0216 ± 0.0015
*A*_4_/J mol^−1^ K^−5^	− 5.2876 × 10^−4^	(− 8.276 ± 0.600) × 10^−4^
*A*_5_/J mol^−1^ K^−6^	8.8622 × 10^−6^	(1.541 ± 0.123) × 10^−5^
*A*_6_/J mol^−1^ K^−7^	− 5.7267 × 10^−8^	(− 1.130 ± 0.010) × 10^−7^

**Fig. 7:**
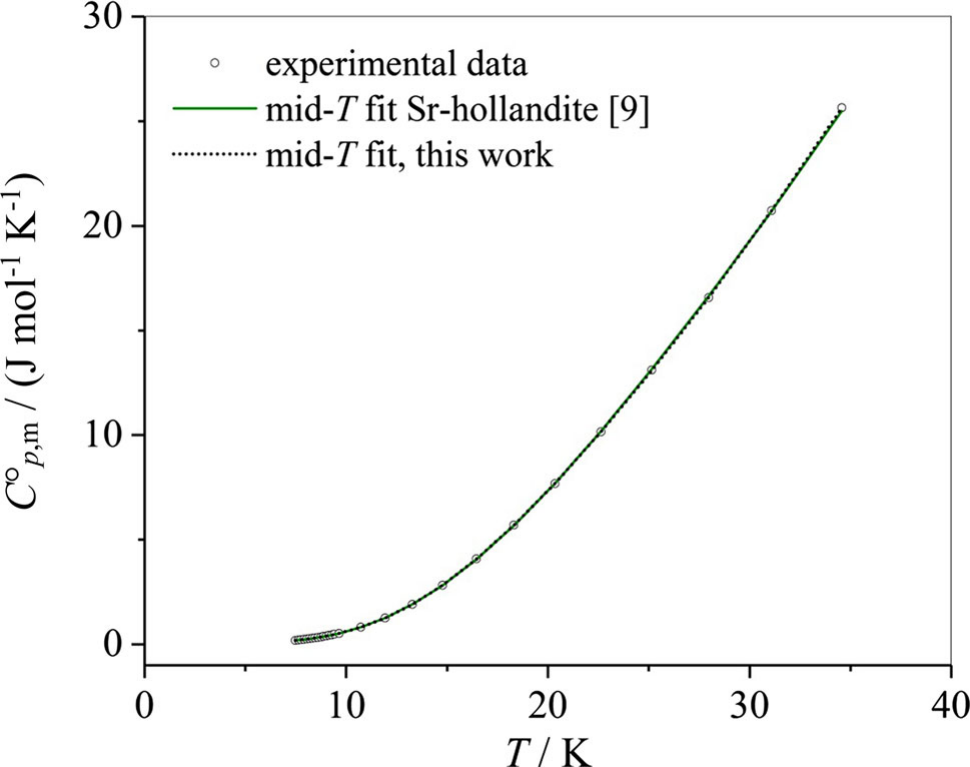
Comparison of the mid-*T* Sr-hollandite fitting curves, which completely overlap

It is demonstrated in Fig. 7 that both polynomial fits (the one from Wu et al. [9] and that calculated within this work) are equivalent.

However, the fit parameters deviate from each other, indicating that the fit parameters for the polynomials are correlated with each other. The error of the fit coefficients is partly not even one order of magnitude smaller than the coefficients.

The goal to simulate the experimental data in a precise way is certainly achieved by this method as it is by using intersecting spline functions. As a preferred alternative, however, it is suggested to determine six semi-empiric fit coefficients of the Debye–Einstein integral for the whole temperature range from zero to approximately 300 K.

**Fig. 8:**
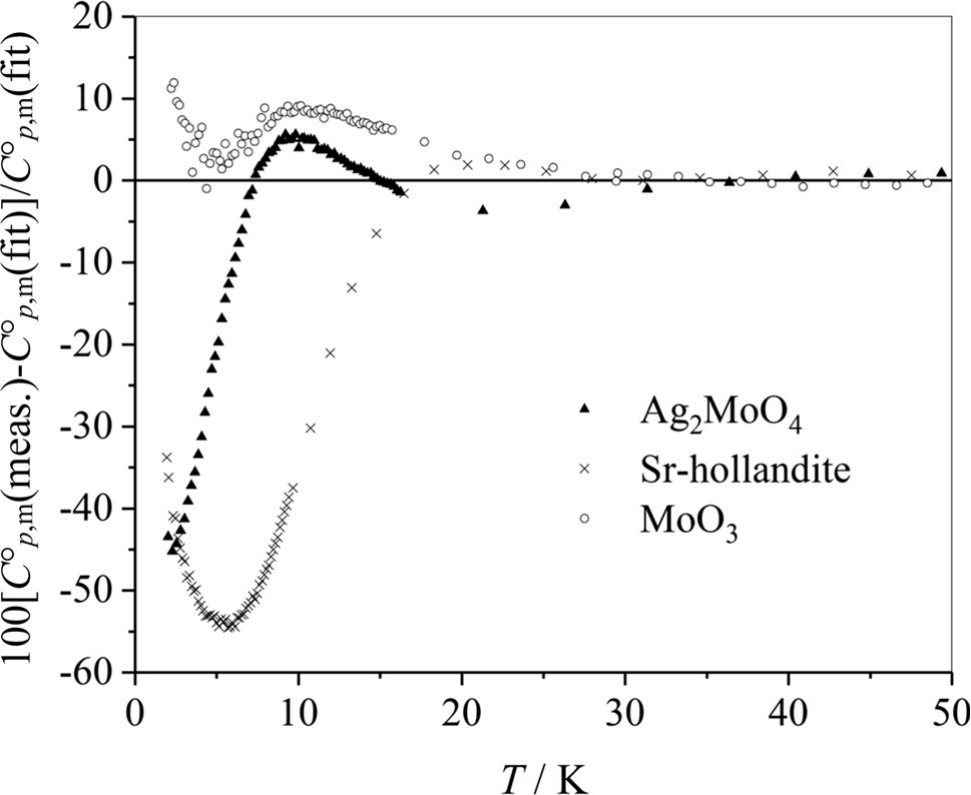
Deviations of the experimental values of the heat capacity from the fitted values

The quality of the six-parameter Debye–Einstein fit is checked for three representative examples—Ag_2_Mo_4_, Sr-hollandite, and MoO_3_—by plotting the relative deviations of the measured heat capacity $C_{{p,{\text{m}}}}^{\text{o}} ({\text{meas}} .)$ values to the fitted heat capacities $C_{{p,{\text{m}}}}^{\text{o}} ({\text{fit}})$ in percent, ${{100\; \times \;\left[ {C_{{p,{\text{m}}}}^{\text{o}} ({\text{meas}} .) - C_{{p,{\text{m}}}}^{\text{o}} ({\text{fit}})} \right]} \mathord{\left/ {\vphantom {{100\; \times \;\left[ {C_{{p,{\text{m}}}}^{\text{o}} ({\text{meas}} .) - C_{{p,{\text{m}}}}^{\text{o}} ({\text{fit}})} \right]} {C_{{p,{\text{m}}}}^{\text{o}} ({\text{fit}})}}} \right. \kern-0pt} {C_{{p,{\text{m}}}}^{\text{o}} ({\text{fit}})}},$ see Fig. 8.

The experimental values deviate from the fit at ultra-low temperatures. However, heat capacities at temperatures of 20 K and higher are described reasonably well.

In case that very precise low temperature data (*T* < 20 K) are required Musikhin et al. [8] suggested a Debye–Einstein fit function (see Eq. (1) in [8]) with three fit coefficients that usually describe the experimental data very well especially when vibrational contributions to the heat capacity are dominant.

Furthermore, it can be tried to increase the number of semi-empiric fit coefficients, e.g., by the eight parameter Debye–Einstein approach:

5 $$C_{{p , {\text{ m}}}}^{\text{o}} = m_{ 1} D(\theta_{\text{D1}} /T) + m_{ 2} D(\theta_{\text{D2}} /T) + n_{ 1} E_{ 1} (\theta_{\text{E1}} /T) + n_{ 2} E_{ 2} (\theta_{{{\text{E}}2}} /T).$$

**Fig. 9:**
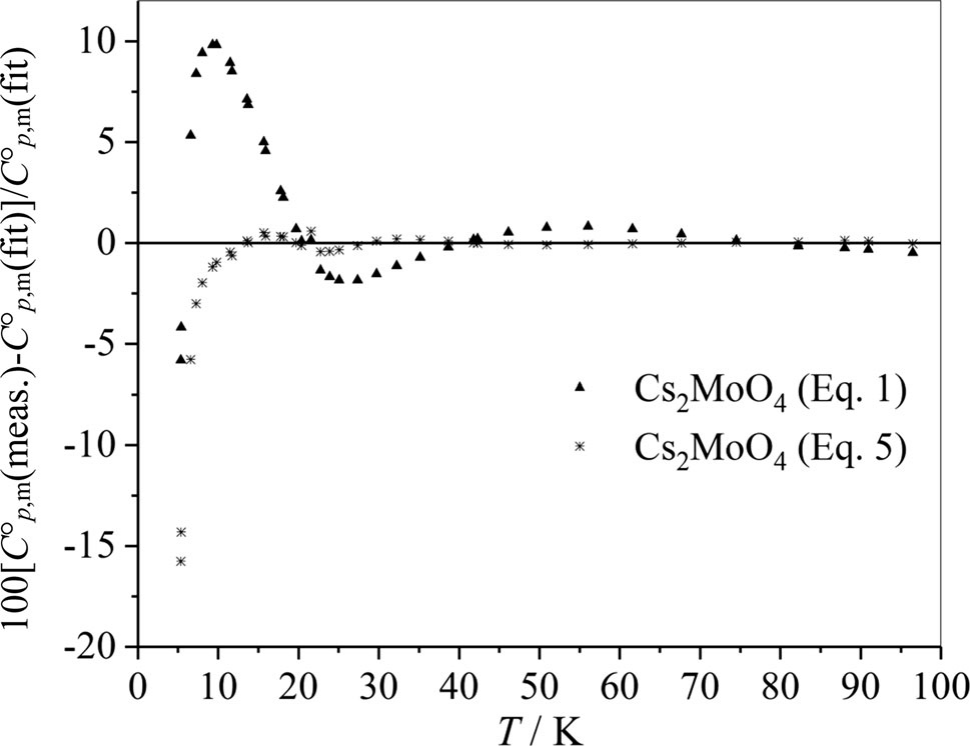
Deviations of the experimental values of the heat capacity from the fitted values for Cs_2_MoO_4_ comparing the six-parameter Debye–Einstein approach (Eq. (1)) with the eight-parameter Debye–Einstein approach

The two-Debye functions improve the low temperature description, whereas in an approach with three Einstein temperatures two Einstein temperatures are usually strongly correlated when the coefficients (*n*_1_, *n*_2_, and *n*_3_) are not constrained. As an example experimental data of the heat capacity of Cs_2_MoO_4_, which can be found in [20], are described by both the six-parameter Debye–Einstein approach (Eq. (1)) and the eight parameter Debye–Einstein fit (Eq. (5)). The fit parameters are summarized in Table 20. The quality of the fitting curves is compared by plotting deviations of the experimental data from the fitted values (Fig. 9). It can be stated again that deviation from the experimental values is very small except for very low temperatures. Deviations of the experimental values from the fitted values are smaller for eight parameter Debye–Einstein fit (Eq. (5)) compared to the six-parameter Debye–Einstein approach (Eq. (1)). However, physics seems to be captured in a better way by the simpler fit, since the sum of the adjusted pre-factors is closer to the number of atoms in the formula unit (7 for Cs_2_MoO_4_ and 7.4 from Eq. (1) versus 8.1 from Eq. (5)).

**Table 20:** Characteristic temperatures *θ*_D_, *θ*_E1_, *θ*_E2_ and adjusted parameters of the heat capacity of the Cs_2_MoO_4_

Characteristic temperatures (Eq. (1))	Value	Adjusted pre-factors (Eq. (1))	Value
*θ*_D_/K	122.8 ± 0.7	*m*	3.620 ± 0.026
*θ*_E1_/K	347.8 ± 9.0	*n*_1_	1.800 ± 0.046
*θ*_E2_/K	1044 ± 37	*n*_2_	1.953 ± 0.045

Characteristic temperatures (Eq. (5))	Value	Adjusted pre-factors (Eq. (5))	Value
*θ*_D1_ */K*	108.3 ± 0.9	*m*_1_	2.777 ± 0.056
*θ*_D2_ */K*	1870 ± 41	*m*_2_	2.443 ± 0.050
*θ*_DE1_ */K*	160.7 ± 3.4	*n*_1_	1.320 ± 0.042
*θ*_DE2_ */K*	461.4 ± 5.7	*n*_2_	1.604 ± 0.016

## Conclusions

It was already shown in Gamsjäger et al. [13] that it is possible to extrapolate heat capacity values for temperatures below 50 K when experimental values are only available at higher temperatures by the Kelley–King approach [16]. It is demonstrated in this work that the low temperature heat capacity up to 300 K of very different compounds can be approximated by the Debye–Einstein integral fit, Eq. (1). It is likely that the simple, however, versatile Debye–Einstein integral fit (Eq. (1)) suffices to approximate the heat capacity of several crystalline compounds between 0 and 300 K in case that no phase transformation occurs in this temperature range. It is thus recommended to apply this simple semi-empiric fit (Eq. (1)) to experimental heat capacity data in a first approach. Whereas the residuals at ultra-low temperatures (below 20 K) might be large, the influence on the thermodynamic functions at elevated temperatures is small as can be seen by comparing the literature data with the results from this work. It is noted that an additional precise description of ultra-low temperature data (0–20 K) is required in case that thermodynamic functions are requested in this ultra-low temperature range. The Debye–Einstein integral fit (Eq. (1)) is advantageous compared to more sophisticated fitting approaches where the fit coefficients lose their physical meaning and might be strongly correlated with each other.

## References

[1] Hwang JS, Lin KJ, Tien C (1997). Rev Sci Instrum.

[2] Bissengaliyeva MR, Gogol DB, Taimassova ST, Bekturganov NS (2012). J Chem Thermodyn.

[3] Bissengaliyeva MR, Gogol DB, Taimassova ST, Bekturganov NS (2012). J Chem Thermodyn.

[4] Morishita M, Houshiyama H (2015). Mater Trans.

[5] Morishita M, Fukushima M, Houshiyama H (2016). Mater Trans.

[6] Morishita M, Houshiyama H, Kinoshita Y, Nozaki A, Yamamoto H (2017). Mater Trans.

[7] Musikhin AE, Naumov VN, Bespyatov MA, Ivannikova NV (2015). J Alloys Compd.

[8] Musikhin AE, Naumov VN, Bespyatov MA, Shlegel VN (2016). J Alloys Compd.

[9] Wu L, Schliesser J, Woodfield BF, Xu H, Navrotsky A (2016). J Chem Thermodyn.

[10] Wu Z, Dreisinger DB, Urch H, Fassbender S (2014). Hydrometallurgy.

[11] De Velasco Maldonadoa PS, Hernández-Montoya V, Concheso A, Montes-Morán MA (2016). Appl Surf Sci.

[12] Boerio-Goates J, Stevens R, Hom BK, Woodfield BF, Piccione PM, Davis MK, Navrotsky A (2002). J Chem Thermodyn.

[13] Gamsjäger E, Morishita M, Gamsjäger H (2016). Monatsh Chem.

[14] Woodfield BF, Boerio-Goates J, Shapiro JL, Putnam RL, Navrotsky A (1999). J Chem Thermodyn.

[15] http://www.maplesoft.com. Accessed June 2017

[16] Kelley KK, King EG (1961) Contributions to data on theoretical metallurgy: XIV. Entropies of the elements and inorganic compounds. Bulletin 592, United States Bureau of Mines

[17] Morishita M, Kinoshita Y, Houshiyama H, Nozaki A, Yamamoto H (2017). J Chem Thermodyn.

[18] http://www.originlab.com. Accessed June 2017

[19] Dachs E, Bertoldi C (2005). Eur J Miner.

[20] Osborne DW, Flotow HE, Hoekstra HR (1974). J Chem Thermodyn.

